# The prevalence and profiles of adverse childhood experiences and their associations with adult mental health outcomes in China: a cross-sectional study

**DOI:** 10.1016/j.lanwpc.2024.101253

**Published:** 2024-12-05

**Authors:** Peilin Xu, Zhaorui Liu, Yifeng Xu, Tao Li, Guangming Xu, Xiangdong Xu, Limin Wang, Yongping Yan, Shuiyuan Xiao, Lingjiang Li, Tingting Zhang, Jie Yan, Yaqin Yu, Xiufeng Xu, Zhizhong Wang, Bo Wang, Wanjun Guo, Yueqin Huang

**Affiliations:** a Peking University Sixth Hospital, Peking University Institute of Mental Health, NHC Key Laboratory of Mental Health (Peking University), National Clinical Research Center for Mental Disorders (Peking University Sixth Hospital), Beijing 100091, China; b Shanghai Mental Health Center, School of Medicine, Shanghai Jiao Tong University, Shanghai 200030, China; c Affiliated Mental Health Center & Hangzhou Seventh People’s Hospital, Zhejiang University School of Medicine, Hangzhou, Zhejiang 310058, China; d Tianjin Anding Hospital, Mental Health Center of Tianjin Medical University, Tianjin 300222, China; e The Fourth People's Hospital in Urumqi, Urumqi 830002, China; f National Center for Chronic and Non-communicable Disease Control and Prevention, Chinese Center for Disease Control and Prevention, Beijing 100050, China; g Department of Epidemiology, The Fourth Military Medical University, Xi’an, Shaanxi 710032, China; h Department of Social Medicine and Health Management, School of Public Health, Central South University, Changsha, Hunan 410078, China; i Mental Health Institute, The Second Xiangya Hospital, Central-south University, Changsha, Hunan 410011, China; j Institute of Social Science Survey, Peking University, Beijing 100871, China; k Department of Epidemiology and Biostatistics, School of Public Health, Jilin University, Changchun, Jilin 130021, China; l Department of Psychiatry, The First Affiliated Hospital of Kunming Medical University, Kunming, Yunnan 650032, China; m Department of Epidemiology and Statistics, School of Public Health and Management, Ningxia Medical University, Yinchuan, Ningxia 750004, China; n Mental Health Center, West China Hospital, Sichuan University, Chengdu, Sichuan 610041, China

**Keywords:** Adverse childhood experiences, Mental health outcomes, Latent class analysis, Population-attributable fractions

## Abstract

**Background:**

Adverse childhood experiences (ACEs) are common and associated with mental disorders. However, the prevalence and co-occurrence of ACEs and their association with mental health outcomes among Chinese adults have not been well demonstrated.

**Methods:**

China Mental Health Survey, a cross-sectional nationally representative survey consisting of 28,140 adults, was conducted from July 2013 to March 2015. Twelve ACEs and mental health outcomes, including mood disorder, anxiety disorder, substance-use disorder, and suicide were measured using the Composite International Diagnostic Interview (CIDI) 3.0 in a weighted representative subsample of 9378 respondents. Latent class analysis was used to identify the co-occurrence profiles of ACEs, and logistic regression was applied to examine the association between ACEs and mental health outcomes. Population-attributable fractions (PAFs) were calculated to quantify the attribution of ACEs to these outcomes.

**Findings:**

Among the 9378 respondents, the weighted count (proportion) of females was 4642 (49.5%), with a weighted mean (SD) age of 43.0 (15.8) years. In this study, 27.1% of respondents showed at least one ACE, with multiple ACEs common (37.6%) among those affected. Neglect was the most prevalent ACE (11.3%), followed by physical abuse (9.1%). Latent class analysis identified four co-occurrence profiles: low risk of ACEs, maltreatment, caregiver’s maladjustment and parental loss. The strongest association with mental health outcomes was found in the caregiver’s maladjustment group (OR, 4.9; 95% CI, 3.2–7.6). Estimates of PAFs indicated that all ACEs together explained 39.4% (95% CI, 31.3%–47.4%) of observed mental health outcomes. Gender differences were noted in prevalence and associations with outcomes.

**Interpretation:**

ACEs are highly prevalent and interrelated in China, attributing to over one-third of the mental disorder burden. In resource-limited settings, prioritizing the reduction of the most prevalent and impactful ACEs through education and policy can more effectively alleviate the disease burden.

**Funding:**

The National Twelfth Five-Year Plan for Science and Technology Support from the Chinese Ministry of Science and Technology (grant numbers 2012BAI01B01 & 2015BAI13B00), and the 10.13039/501100012166National Key R&D Program of China (grant numbers 2017YFC0907800 and 2017YFC0907801).

**Research in context**

**Evidence before this study**

We searched PubMed for articles published from database inception to July 10, 2024, using a combination of terms: ((“adverse childhood experience” OR “childhood adversity” OR “childhood trauma” OR “childhood abuse”) AND (“mental disorder” OR “mood disorder” OR “anxiety disorder” OR “substance-use disorder” OR “suicide” OR “psychopathology”)). We further searched EMBASE and Google Scholar using similar terms. Most of these studies conducted in developed country or Chinese specific populations and have ignored the co-occurrence of ACEs. Besides, association studies mostly relied on scales or self-developed questionnaires to assess mental health outcomes. The prevalence and co-occurrence of ACEs and their associations with mental health outcomes in China remain unknown.

**Added value of this study**

We used data from China Mental Health Survey, the first and only nationally representative community survey on mental health in China, employed standardized assessment and diagnostic procedure. Over a quarter (27.1%) of Chinese adults have experienced at least one ACE, with neglect being the most common type. We identified four distinct ACE profiles, with the caregiver’s maladjustment profile showing the strongest association with any mental health outcomes. A total of 39.4% observed mental health outcomes were attributable to ACEs in China. Among individual ACE types, caregiver’s mental disorders had the highest population attributable fraction.

**Implications of all the available evidence**

This study highlights the importance of parenting education in addressing modifiable ACEs, especially neglect and physical abuse, which are prevalent and strongly associated with mental health outcomes. Moreover, ACEs assessments could serve as valuable indicators for mental disorder screening, particularly for unmodifiable ACEs. Early identification of this ACE and interventions of providing social support and strengthening psychological resilience may reduce the risks for mental disorders. Given the co-occurrence of ACEs, addressing common factors is essential for effectively reducing their prevalence and associated mental health issues, particularly in resource-limited settings.

## Introduction

Adverse childhood experiences (ACEs) refer to a wide range of difficult, unpleasant situations or stressful experiences occur in childhood.^1^ According to a meta-analysis of 206 studies, representing 546,458 adult participants across 22 countries, 60.1% showed having experienced at least one ACE, and more than half of them showed multiple adversities.^2^ During the highly malleable period of psychological development, ACEs can affect the function of stress response systems, the hypothalamic–pituitary–adrenal (HPA) axis, and neurocognition,^3^ leading to an increased risk of mental disorders and suicide.^4^, ^5^, ^6^, ^7^

The epidemiological characteristics and impacts of ACEs vary across socio-economic statuses and races.^2^ Understanding the prevalence of ACEs and their association with mental health outcomes in a specific country helps to understand the disease burden caused by ACEs and identify the priority intervention types at the societal level. In traditional Chinese culture, people generally follow strict childrearing practices, including the parental authority, child obedience, and family harmony. Therefore, China may have distinctive prevalence and co-occurrence of ACEs and the associations with mental health outcomes, especially in the context of changing parenting practices following the cancel of one-child policy.

However, several limitations hinder the clarification of this critical issue. First, previous studies in China were conducted in regional or age-specific populations, which may not accurately represent the prevalence of ACEs among Chinese adults across a broad range of ages and regions.^8^, ^9^, ^10^, ^11^, ^12^, ^13^ Moreover, most studies simply summed up a cumulative index of ACEs, overlooking the interrelation and co-occurrence of these experiences.^5^^,^^14^, ^15^, ^16^, ^17^, ^18^ Nevertheless, scales or self-developed questionnaires were commonly used to assess symptoms of mental disorders.^11^, ^15^, ^19^, ^20^, ^21^ Lacking standardized diagnostic procedures, the reliability of their results on the associations between ACEs and mental disorders may be compromised. Gender inequities may lead to a greater susceptibility to certain ACEs for one gender, while differences in developmental processes and brain function lead to diverse psychophysiological responses to ACEs exposure.^22^ However, current evidence on gender differences in ACEs remains inconsistent.^23^^,^^24^

China Mental Health Survey (CMHS) was the first and only nationally representative community survey on mental disorder and utilization of health service in China. Applying a complex multi-stage sampling and weighting process, the sample's characteristics demonstrated a good fit with the overall Chinese population.^25^ Based on the CMHS, this study aimed to estimate the prevalence and co-occurrence profiles of ACEs and their associations with mental health outcomes in China.

## Methods

### Sample

The study subjects were respondents from the CMHS, a cross-sectional epidemiological survey on mental disorders conducted from July 22, 2013 to March 5, 2015 in China. The sample included individuals aged 18 and older across the mainland of China (excluding Hong Kong, Macau, and Taiwan). To achieve a nationally representative sample, the CMHS utilized the Disease Surveillance Points system, managed by the Chinese Center for Disease Control and Prevention (CDC), which included 157 surveillance points covering both urban district and rural county in China. Using a multistage, population-proportional sampling method, the CMHS identified 40,964 households across 1256 communities or villages, distributed in 628 streets or towns from 157 sampling sites. After excluding empty households or invalid addresses, the Kish table sampling was then employed to randomly select one respondent from the eligible household members.

Briefly, a sample of 28,140 respondents was face-to-face interviewed by trained lay-interviewers and completed Part I interview to assess the presence of mental disorders. Those who met criteria for at least one mental disorder in the Part I interview, along with 25% of those who did not meet these criteria, were simultaneously included in the Part II interview (n = 9378), which included questions about in-depth demographics, potential risk factors, and ACEs. This study was based on data from this Part II subsample, with final weights accounting for both the multi-stage sampling design and the selection of Part II respondents from Part I (Detailed in eFig. 1 in Supplement). The demographic characteristics of the sample were consistent with those of the national census.^26^ The further description of detailed sampling methodology and weighting procedure of the CMHS was published elsewhere.^27^^,^^28^

The research protocol of the CMHS was approved by the ethics committee of the Sixth Hospital of Peking University in Beijing, China (IMH-IRB-2013–13–1), and all respondents provided written informed consent forms.

### Measures

Mental disorders diagnoses were based on the Composite International Diagnostic Interview (CIDI) 3.0, a fully structured clinical diagnostic interview assessment, which could be used by trained non-professionals in face-to-face interviews.^29^ CIDI-3.0 was specifically designed with consideration for the sensitive nature of mental health information and has been widely used in studies of ACEs worldwide.^30^ It mandates the use of standardized clarification and probing techniques by interviewers, along with the implementation of motivational instructions, commitment questions, contingent reinforcement and confidentiality notification, all of which effectively enhance the validity of data collection.^29^ The reliability and validity of the Chinese version of CIDI-3.0 were evaluated in China.^31^

Based on this standardized instrument, mental disorders were retrospectively assessed by applying a diagnostic algorithm based on the Diagnostic and Statistical Manual of Mental Disorders, Fourth Edition (DSM-IV). This study examined mood disorder (depressive disorder, bipolar disorder, substance-induced mood disorder and mood disorder due to a general medical condition), anxiety disorder (generalized anxiety disorder, panic attack, post-traumatic stress disorder, agoraphobia without panic disorder, social phobia, specific phobia, obsessive compulsive disorder, substance-induced anxiety disorder, anxiety disorder due to a general medical condition, and anxiety disorder not otherwise specified), substance-use disorder (alcohol use disorder and drug use disorder) and suicide (ideation, plans and attempts).

Twelve ACEs were assessed in a separate section about childhood experience in CIDI-3.0,^23^ based on the respondent's self-reported occurrence of such experiences during childhood, including parental death, parental divorce, other parental loss, caregiver’s mental disorder, caregiver’s substance abuse, caregiver’s criminality, caregiver’s violence, physical abuse, sexual abuse, neglect, serious physical illness, and family economic adversity. The measures of parental death, divorce, and other separation were focused on biological parents, not step-parents or other caregivers. The measures of caregiver’s mental disorder and substance abuse included major depression, generalized anxiety disorder, panic disorder, alcohol and drug misuse and dependence. Caregiver’s criminality was assessed with questions about whether a caregiver either engaged in criminal activities like burglary or stealing property or was ever arrested for crime. Caregiver’s violence was assessed with questions about whether a caregiver often engaged in physical fights. Physical abuse was assessed based on the question about whether had been regularly pushed, pinched, hit, or had things thrown at body. Sexual abuse was assessed with questions about whether had been repeatedly fondled, rape or attempted rape. Neglect was assessed with the frequency of having inadequate food, clothing, or medical care and supervision despite these resources being available, and having to do age-inappropriate chores. As structural stressors and potential causes of other ACEs,^32^ family economic adversity and serious physical illness were included and assessed separately through questions about whether a family received government assistance for an extended period and whether the respondent experienced life-threatening illness during childhood. Neglect and physical abuse were measured on an ordinal scale with response options ranging from “never,” “rarely,” “sometimes,” to “often,” with the latter two categories classified as positive.^29^^,^^30^^,^^33^ All other types were assessed using binary (yes/no) responses (Detailed in the Supplement). Then, the total number of ACE categories was summed to create the number of ACEs ranging from 0 to 10.

### Statistical analysis

The weighted prevalence of 12 ACEs was described to represent the proportion of all respondents who experienced any ACEs in China. Weighting methods, including sampling design, non-response adjustment, and post-stratification adjustment were employed and published elsewhere.^28^ Latent class analysis (LCA) was performed to identify distinct patterns of ACEs in China based on their co-occurrence. LCA is a clustering technique that identifies unobserved but statistically distinct and clinically meaningful subpopulations based on how they respond to observed categorical variables using maximum likelihood estimation.^34^^,^^35^ Models were estimated with 1–5 patterns, evaluating fit indices such as the Akaike Information Criterion (AIC), Bayesian Information Criterion (BIC), sample size adjusted BIC (SSABIC), and entropy values. Lower AIC, BIC and SSABIC scores and higher entropy values indicate more accurate classification and a more optimal model.^36^ Ultimately, the best model was selected based on a balanced evaluation of fit indices, practical value, and conceptual criteria.

Differences in categorical sociodemographic variables were examined across respondents with and without ACEs and among different ACE profiles using the χ^2^ test. Logistic regression models were established to assess the associations of types, number, and profiles of ACEs with mental health outcomes, including mood disorder, anxiety disorder, substance disorder, and suicide. Independent variables included any single ACE, the number of ACEs, and ACE profiles. The models were adjusted for gender, age, years of education, marital status, urban-rural residence, region, working status and ethnic group (Han vs. non-Han Chinese), referring to previous studies.^37^^,^^38^ Odds ratios (ORs) and 95% confidence intervals (CIs) were presented. Population-attributable fractions (PAFs) were calculated for significant associations to quantify the attribution of ACEs to mental health outcomes, with CIs generated using the bootstrap method.

Gender-specific findings for the prevalence of ACEs and their main associations with mental health outcomes were reported. The heterogeneity of associations among subgroups was tested using Cochran's Q test.

All analyses were conducted using SAS version 9.4, along with the LCA extension (The Methodology Center). All p values were derived from 2-sided tests, and results were considered statistically significant at p < 0.05.

### Role of the funding source

The funders of the study had no role in the study design, data collection, data analysis, data interpretation, or writing of the report.

## Results

Among the 9378 respondents who completed the ACEs-related interview in Part II, the weighted mean (SD) age was 43.0 (15.8) years, with a weighted count (proportion) of 4642 (49.5%) for females and 4855 (51.8%) for urban residents. Overall, 2952 (weighted prevalence, 27.1%) of the respondents showed exposure to at least one ACE (Table 1). The most prevalent ACE was neglect (1161 [weighted prevalence, 11.3%]), followed by physical abuse (919 [weighted prevalence, 9.1%]). Among respondents with any ACEs, multiple ACEs were common (weighted proportion, 37.6%).

**Table 1:** Prevalence of adverse childhood experiences in China.

Type of ACEs	N	Weighted	
		N	Prevalence % (S.E)
Parental divorce	100	64	0.7 (0.1)
Parental death	393	285	3.1 (0.3)
Other parental loss	518	429	4.6 (0.4)
Caregiver’s substance abuse	131	121	1.3 (0.2)
Caregiver’s criminality	36	32	0.3 (0.1)
Caregiver’s violence	43	28	0.3 (0.1)
Caregiver’s mental disorders	924	668	7.3 (0.6)
Neglect	1161	1050	11.3 (0.9)
Physical abuse	919	850	9.1 (0.7)
Sexual abuse	10	2	0.1 (0.1)
Family economic adversity	179	159	1.7 (0.2)
Serious physical illness	339	175	1.9 (0.2)
Any adverse childhood experiences	2952	2541	27.1 (1.2)
Total number of ACEs			
1	1759	1586	62.4 (1.9)
2	767	687	27.0 (1.9)
3	299	190	7.5 (0.9)
≥4	127	77	3.0 (0.7)

ACEs, Adverse childhood experiences; S. E., standard error.

**Fig. 1:**
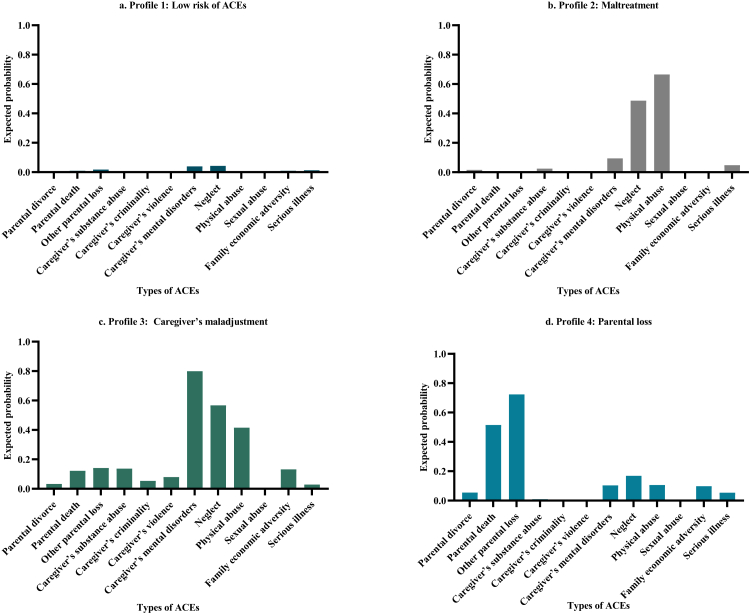
Latent Profiles of ACE Indicator. Each figure represents a different adverse childhood experience profile.

Following a thorough examination of the fit indices and the substantive meaning of the latent profiles, the four-profile solution was deemed optimal (Fig. 1, eTable 1 in Supplement). Profile 1, the largest group (weighted proportion, 86.7%), exhibited low probabilities of experiencing ACEs. It served as the baseline and was labeled the “low risk of ACEs” profile. Profile 2 (weighted proportion, 8.3%) showed a low risk of experiencing most adversities except neglect or physical abuse. It was labeled the “maltreatment” profile. Profile 3 (weighted proportion, 2.5%) exhibited a high probability of caregiver’s mental disorder, substance abuse, criminality, and violence. It was labeled the “caregiver’s maladjustment” profile. Profile 4 (weighted proportion, 2.5%) was characterized by experiencing a wide range of ACEs, particularly parental separation due to various reasons and was labeled the “parental loss” profile.

Socio-demographic characteristics varied across respondents with different ACEs exposure (eTables 2 and 3 in Supplement). The maltreatment and caregiver’s maladjustment profiles had a higher proportion of males. Besides, respondents in the parental loss profile were older and had fewer education years than the other three profiles, and more were never married than the low risk of ACEs profile.

**Fig. 2:**
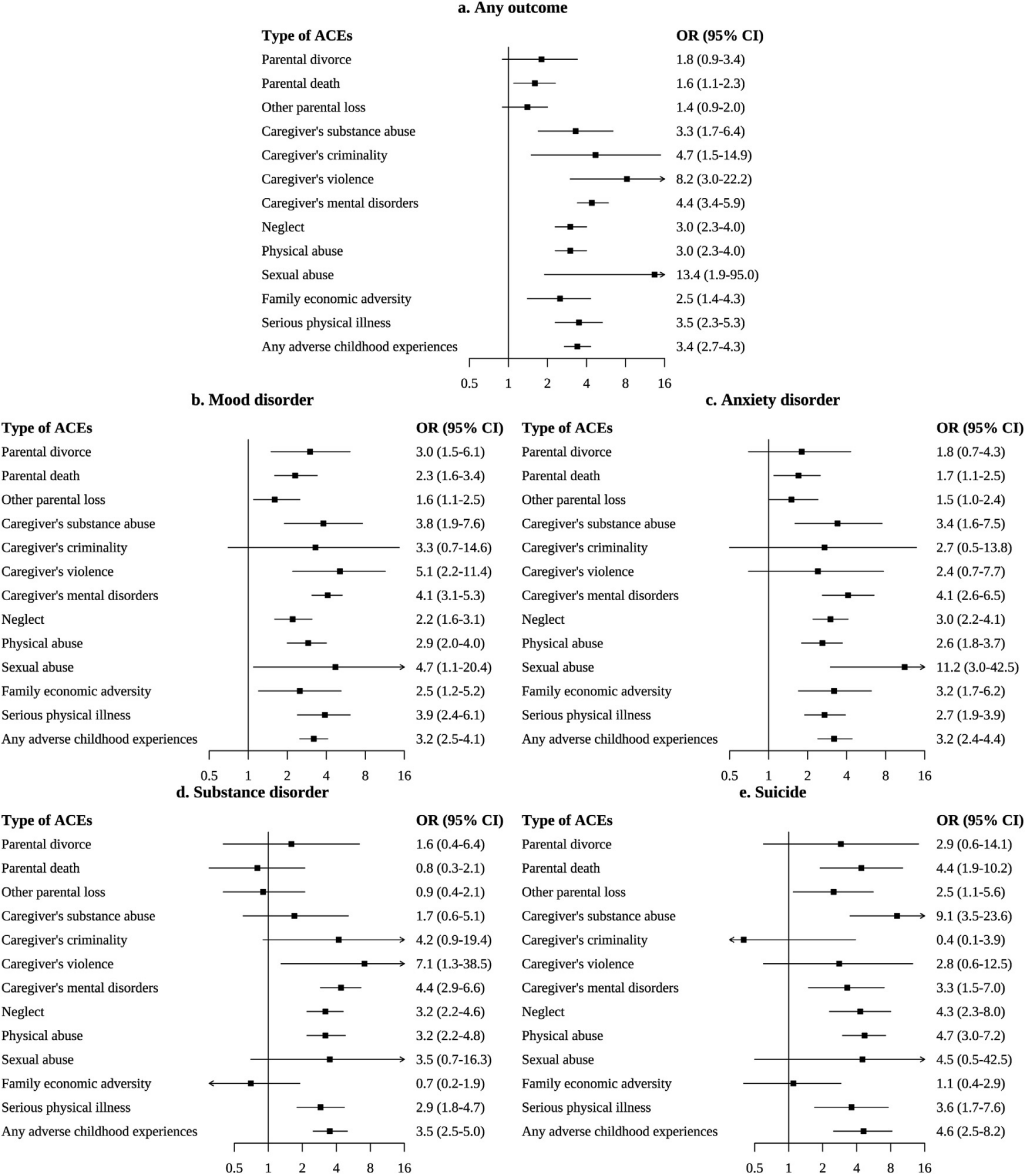
The Association Between Types of ACEs and Mental Health Outcomes. Abbreviations: ACEs, adverse childhood experiences; OR, odds ratio; CI, confidence interval. Models were adjusted for gender, age, rural or urban residence, education level, marital status, region, working status and ethnic group. Odds ratios (ORs) represent the association between ACEs and mental health outcomes. Error bars indicate 95% CIs.

Compared with individuals without ACE exposure, those who experienced any ACE had increased risks of mental health outcomes (OR, 3.4; 95% CI, 2.7–4.3) (Fig. 2). Neglect and physical abuse, the two most prevalent types of ACEs, were strongly associated with mental health outcomes, particularly suicide (Neglect: OR, 4.3; 95% CI, 2.3–8.0; Physical abuse: OR, 4.7; 95% CI, 3.0–7.2). Additionally, caregiver’s mental disorder and serious physical illness were also associated with all four mental health outcomes. Moreover, the number of ACEs was consistently associated with the increased risk for all four mental health outcomes and presented a clear dose–response gradient in the association with substance-use disorder and suicide. However, these trends were only observed in the association with any mental health outcomes, mood disorder, and anxiety disorder when the number did not exceed 3. Furthermore, compared with the low risk of ACEs profile, the other three profiles were significantly associated with an increased risk of mental health outcomes. The caregiver’s maladjustment profile exhibited the strongest associations with mental health outcomes (OR, 4.9; 95% CI, 3.2–7.6), as well as mood disorder (OR, 5.1; 95% CI, 3.2–8.3) and anxiety disorder (OR, 5.5; 95% CI, 3.3–9.2). In addition, three profiles had similar estimated effect of the associations with suicide, the estimated ORs range from 4.1 (95% CI, 1.7–9.9) to 4.3 (95% CI, 2.3–7.8). Only maltreatment profile (OR, 3.3; 95% CI, 2.2–5.1) and caregiver’s maladjustment profile (OR, 2.5; 95% CI, 1.1–5.7) were associated with substance-use disorder compared with the low risk of ACEs profile (Table 2).

**Table 2:** The association between multiple ACEs and mental health outcomes (OR (95% CI)).

Multiple ACEs	Mood disorder	Anxiety disorder	Substance-use disorder	Suicide	Any outcome
Number of ACEs^a^					
1	2.6 (1.9–3.5)	2.7 (1.9–3.7)	3.1 (2.0–4.9)	3.1 (1.6–6.0)	2.9 (2.3–3.7)
2	3.2 (2.2–4.8)	3.3 (2.2–5.0)	3.5 (2.1–5.8)	5.2 (2.4–11.7)	3.7 (2.6–5.2)
3	8.3 (5.2–13.2)	8.4 (5.1–13.9)	6.0 (3.8–9.4)	9.7 (4.1–23.0)	8.5 (5.8–12.4)
≥4	6.9 (3.2–14.7)	5.6 (2.8–11.3)	6.5 (2.2–18.8)	20.9 (5.7–76.9)	6.3 (2.5–15.7)
Profile of ACEs^b^					
Profile 2: maltreatment	2.8 (2.0–3.9)	2.4 (1.6–3.4)	3.3 (2.2–5.1)	4.3 (2.3–7.8)	3.0 (2.3–4.1)
Profile 3: caregiver's maladjustment	5.1 (3.2–8.3)	5.5 (3.3–9.2)	2.5 (1.1–5.7)	4.1 (1.7–9.9)	4.9 (3.2–7.6)
Profile 4: parental loss	2.6 (1.6–4.1)	2.0 (1.3–2.9)	0.7 (0.3–1.4)	4.1 (2.1–8.0)	1.8 (1.1–2.9)

ACEs, adverse childhood experiences; OR, odds ratio; CI, confidence interval.

Models were adjusted for gender, age, rural or urban residence, education level, marital status, region, working status and ethnic group.

a Reference: No ACE exposure.

b Reference: Profile 1 (Low risk of ACEs).

ACEs had similar attribution of mood disorder (PAF, 37.3; 95% CI, 28.6–46.0), anxiety disorder (PAF, 37.3; 95% CI, 26.7–47.6), and suicide (PAF, 40.4; 95% CI, 28.4–51.7), with a greater attribution observed in substance use disorder (PAF, 49.4; 95% CI, 29.0–66.4) (Table 3). Overall, ACEs accounted for 39.4% (95% CI, 31.3%–47.4%) of the observed mental health outcomes in all respondents. Among these, caregiver’s mental disorder attributed the highest proportion (PAF, 19.8; 95% CI, 14.0–26.1).

**Table 3:** Population attributable fraction of ACEs for four mental health outcomes ((95% CI), %).

Type of ACEs	Any outcome	Mood disorder	Anxiety disorder	Substance-use disorder	Suicide
Parental divorce	NA	1.4 (0.3,3.4)	NA	NA	NA
Parental death	1.8 (0.3,3.9)	3.8 (1.7,7.0)	2.1 (0.4,4.7)	NA	9.4 (2.6,22.3)
Other parental loss	NA	2.7 (0.3,6.2)	NA	NA	6.4 (0.4,17.7)
Caregiver's substance abuse	2.9 (0.9,6.9)	3.5 (1.1,8.4)	3.0 (0.7,8.2)	NA	9.5 (3.0,23.4)
Caregiver's criminality	1.2 (0.1,4.7)	NA	NA	NA	NA
Caregiver's violence	2.1 (0.4,6.6)	1.2 (0.3,3.5)	NA	1.8 (0.1,11.5)	NA
Caregiver's mental disorders	19.8 (14.0,26.1)	18.4 (13.0,24.5)	18.4 (10.3,29.1)	19.8 (12.1,29.6)	14.3 (3.6,30.6)
Neglect	18.5 (12.2,25.8)	12.0 (6.0,19.2)	18.5 (11.7,26.6)	19.9 (11.7,29.4)	27.2 (13.0,45.0)
Physical abuse	15.4 (10.1,21.7)	14.7 (8.6,22.3)	12.7 (6.7,20.1)	16.7 (9.4,25.5)	25.2 (15.2,36.7)
Sexual abuse	0.3 (0.1,2.2)	0.1 (0.1,0.5)	0.2 (0.1,1.0)	NA	NA
Family economic adversity	2.5 (0.7,5.5)	2.5 (0.3,6.8)	3.6 (1.1,8.2)	NA	NA
Serious illness	4.5 (2.3,7.8)	5.1 (2.4,9.3)	3.1 (1.5,5.2)	3.4 (1.4,6.8)	4.6 (1.2,11.4)
Any adverse childhood experiences	39.4 (31.3,47.4)	37.3 (28.6,46.0)	37.3 (26.7,47.6)	40.4 (28.4,51.7)	49.4 (29.0,66.4)

ACEs, adverse childhood experiences; CI, confidence interval; NA, not available.

PAFs were only calculated in the significant associations between ACEs and mental health outcomes.

Compared with females, males were at higher risk of experiencing physical abuse in childhood (10.8% vs. 7.4%, p < 0.05). Among those who had experienced at least one type of ACEs, males had a higher risk of experiencing two types of ACEs (7.9% vs. 5.8%, p < 0.05). Specifically, males were more likely to be classified into maltreatment profile (9.8% vs. 6.7%, p < 0.01) (eFig. 2 in Supplement).

Gender differences were observed in certain associations (eFig. 3 and eTable 5 in Supplement). Males with caregiver’s criminality (OR, 4.5; 95% CI, 1.0–20.8) and caregiver’s violence (OR, 4.0; 95% CI, 1.0–17.1) and family economic adversity (OR, 6.9; 95% CI, 3.0–15.5) in childhood had increased odds of anxiety disorder. Additionally, males with serious physical illness in childhood had increased odds of substance-use disorder (OR, 3.2; 95% CI, 2.0–5.3), and males with caregiver’s substance abuse had increased odds of suicide (OR, 14.4; 95% CI, 4.7–44.0). However, these associations were not significant in females. Moreover, males in maltreatment profile had higher odds of mood disorder (OR, 3.2; 95% CI, 2.2–4.9) and suicide (OR, 7.4; 95% CI, 3.0–18.6) compared with low risk of ACEs profile, although these associations were also significant among females (Of mood disorder: OR, 2.3; 95% CI, 1.5–3.3; Of suicide: OR, 2.6; 95% CI, 1.3–5.1).

## Discussion

Using the data from the CMHS, the first nationally representative survey on mental disorders in China, this study revealed that 27.1% of Chinese adults had at least one ACE and the most prevalent ACE was neglect, followed by physical abuse. Most types of ACEs were associated with mental health outcomes. Among four co-occurrence profiles of ACEs identified during the analysis, caregiver’s maladjustment had the strongest association with mental health outcomes. A total of 37.5% of mental health outcomes could be attributable to ACEs. These findings implicate the widespread prevalence and interconnected nature of ACEs in China, emphasizing the significant association of caregiver’s maladjustment and mental health outcomes.

The World Mental Health (WMH) survey-metropolitan China conducted in Beijing and Shanghai in 2002 using an identical survey procedure and ACEs assessment method, estimated the prevalence of ACEs was 31.0%,^11^ which was higher than the findings of current study. Possible explanations include the greater awareness of ACEs among Chinese metropolitan residents, which may facilitate easier identification. Conversely, rural residents might perceive ACEs differently due to stronger family and community networks.^39^ However, this comparison requires caution due to differences in period and regional sampling. Besides, the China Health and Retirement Longitudinal Study (CHARLS) follow-up surveys, which included respondents aged 45 and older and conducted in 2014–2015, revealed that 80.9% of respondents reported experiencing at least one ACE.^9^ Several university-based studies in China reported the prevalence of ACEs from 43.9% to 86.6%.^8^^,^^10^ The discrepancy may be attributed to differences in the sample’s age composition and the measurement methods. Specifically, their assessment of ACEs relied on questionnaires instead of interviews, and their definitions included a broader range of factors beyond traditional ACEs. For example, CHARLS expanded the concept of community-level ACEs to encompass neighborhood violence and unsafe communities, while university-based research also considered school-related ACEs.

In the horizon comparison, the prevalence of ACEs in China was lower than the results of most studies conducted in other countries with same investigation instrument.^14^^,^^38^^,^^40^, ^41^, ^42^ The WMH Survey, covering 21 countries across different income levels, reported a prevalence of ACEs at 38.8%.^30^ This discrepancy can be partly attributed to the unique cultural perspective in China, where enduring hardships and exhibiting heightened tolerance towards adversities are valued.^43^ Consequently, many ACEs may not be considered as such, and only severe ACEs are likely to be perceived and reported. Notably, the prevalence of specific ACEs may also reflect the unique characteristics of Chinese society. Neglect, the most prevalent type of ACEs in China, is less prominent in other countries,^14^^,^^38^ possibly due to the strict parenting styles in China, which could lead to emotional neglect from the children's perspective.^44^ Additionally, during the early years in China, limited economic conditions forced parents to prioritize work, leaving little time to spend with their children. Furthermore, the prevalence of caregiver’s substance abuse in China was significantly lower than those in other countries. The causes include a deficiency of aldehyde dehydrogenase in Chinese population resulting in alcohol abstinence, China’s strict regulation of drugs^25^ and the older peak age of alcohol use disorder.^45^ Notably, the first Chinese Women's Social Status Survey in 1990 reported a 0.94% prevalence of physical domestic violence against women,^46^ supporting the low prevalence of caregiver’s violence in our study. Besides, the prevalence of family economic adversity in this study was lower than developed country.^14^^,^^47^ This difference may be attributed to most respondents growing up during a period characterized by a collective economy in China, which likely limited their perception of poverty. In this study, respondents whose family received government assistance due to reasons such as loss of labor capacity or disability during childhood were identified as ACE. This perceptible level of extreme poverty may be more significant in identifying the populations at high risk for mental disorders.

In addition to confirming the association between ACEs and mental disorders consistent with previous research,^48^^,^^49^ it was observed that most associations between ACEs and mental health outcomes were higher than those shown in other countries,^33^ which may be related to traditional Chinese cultural norms. The Chinese cultural belief in keeping family issues private may hinder help-seeking and emotional expression among children with ACEs, which is crucial for addressing the onset and development of psychological problems.^50^ On the other hand, Chinese respondents might tend to merely perceive and report severe ACEs, potentially yielding stronger association compared to the results of other countries.^51^ Using LCA, four mutually exclusive and homogeneous profiles were revealed. Caregiver’s maladjustment, which is mainly composed of caregiver’s mental disorder, showed the strongest association among mood disorder and anxiety disorder.^52^^,^^53^ Beyond genetic factors, an unsupportive family environment with caregiver’s mental disorder can disrupt the development of attachment relationships and emotion regulation, thereby resulting in mood and anxiety disorder.^54^ Conversely, maltreatment profile, which is primarily characterized by physical abuse, showed the strongest association with substance-use disorders. Previous studies have indicated that physical abuse leads to persistent alterations in HPA axis in children, affecting their autonomic nervous system responses to stressors^55^ and increasing their likelihood of using psychoactive substances to release the life stress.^56^ However, while many prior studies have reported a stronger association between maltreatment and suicide,^57^^,^^58^ this study revealed that all three ACEs profiles exhibited a similar association with suicide. Due to interpersonal difficulties caused by the parental loss and caregiver’s maladjustment, these children often struggle to access sufficient social support to alleviate their desperation and even suicide in difficult situations later in life.^59^^,^^60^ The findings of LCA provide an opportunity to address multiple ACEs by targeting common underlying causes.

Compared to association metrics, PAF could provide more information on public health significance. Over one-third of mood disorders, anxiety disorders, substance-use disorders, and suicides could be attributable to ACEs in China. This estimate aligns with the findings that all types of ACEs together explained 38.5% of mood disorder, anxiety disorder, substance-use disorder and impulse-control disorder reported in the WMH survey-metropolitan China.^11^

Furthermore, this study found that males had a higher prevalence of physical abuse and exhibited stronger associations between childhood maltreatment and mood disorder than females. One possible explanation is that Chinese parents often hold higher expectations for their sons, leading to stricter discipline and increased physical punishment compared to daughters.^61^ These repeated and uncontrollable maltreatment experiences persisting throughout childhood may contribute to acquired helplessness and poor emotion regulation strategies, ultimately resulting in mood disorder.^62^ Neuroimaging studies have shown gender differences in neural responses to threat, potentially contributing to the observed gender disparities in the association between ACEs and mental health outcomes.^22^^,^^23^ Examining gender-specific ACE prevalence within cultural contexts is essential for understanding and addressing these differences through targeted interventions. However, the directionality of this gender disparity remains unclear, requiring further investigation into the complex associations between gender, ACEs, and mental health outcomes.^24^

### Strengths and limitations

The study had several strengths. Firstly, it employed a nationally representative sample of the Chinese community population and implemented rigorous quality control measures to ensure the validity and reliability of the data, which could firstly provide the valid and reliable results of prevalence and profiles of ACEs unique to China. Secondly, this study utilized the structured diagnostic instrument CIDI 3.0, which follows standardized diagnostic procedures, ensuring enhanced reliability and validity in assessing mental health outcomes compared to screening questionnaires and supporting cross-country comparability.^29^, ^30^, ^31^ Finally, LCA is a better analysis method to identify co-occurrence patterns and characteristics of ACEs among Chinese individuals. Importantly, while ACEs cannot be directly inferred as the full explanation for mental disorders, our findings provide an opportunity to identify high-risk populations for these disorders.

This study has some limitations. Firstly, the informations on ACEs and mental health outcomes were collected retrospectively. Recall bias was inevitable and imbalanced between participants with and without mental health outcomes,^63^^,^^64^ leading to overestimate or underestimate the prevalence of ACEs and its association with the outcomes. Nevertheless, previous studies have demonstrated that the recall of objective events, such as parental death, parental divorce, and caregiver’s criminality, was highly accurate and reliable.^65^^,^^66^ Additionally, meta-analysis suggested that studies using interviews could effectively reduce recall bias compared to questionnaires by providing explanatory context and encouraging dialogue.^66^ Importantly, the ACEs that remain prominent in adult memory may have a lasting impact on well-being. Secondly, the respondents may conceal ACEs for embarrassment, social stigma, and traditional Chinese belief that family shame should not be exposed. Even though interviewers were trained to apply motivational prompts and confidentiality assurances, reporting bias may still be unavoidable. Moreover, neglect and other subjective ACEs may be influenced by the psychological state of the respondents, and existing psychological dysfunction in individuals with mental disorders may result in negative recollection bias.^67^ Moreover, given the basis of observational study, the results should be interpreted with caution on causality. Last, the study included general community residents, leading to limited extrapolation to other population.

Our study has several implications. First, appropriate parenting education should be widely implemented to help address modifiable ACEs, especially neglect and physical abuse, which have high prevalence and strong associations with mental health outcomes. Future efforts should prioritize exploring and evaluating the effective approaches to improve parenting practices. Second, assessments of ACEs could serve as valuable indicators for screening risks of mental disorder, particularly for unmodifiable ACEs. For example, caregiver’s mental disorders may predispose children to genetic vulnerabilities, while early identification of this ACE and interventions aimed at providing social support and strengthening psychological resilience may reduce the risks of mental disorders. Lastly, our latent class analysis suggests that various ACEs may have underlying shared causes. Further research on identifying and addressing common factors could help effectively reduce the prevalence of ACEs and associated mental disorders, particularly in resource-limited settings.

### Conclusions

Overall, it was estimated that 27.1% of Chinese adults have at least one ACE, with neglect and physical abuse being the most prevalent types. This study identified four distinct profiles of ACEs among Chinese population, with the profiles of caregiver’s maladjustment showing the strongest association with mental health outcomes. Approximately one-third of the observed mental health outcomes can be attributed to ACEs. These findings suggest that in resource-limited settings, prioritizing the reduction of the most prevalent and impactful ACEs through health education and policy initiatives, and targeting common underlying factors of co-occurring ACEs, can more effectively alleviate the burden of mental disorders, especially during the transition following the abolition of the one-child policy.

## Contributors

All authors contributed to the collection and interpretation of data, and all authors had final responsibility for the decision to submit for publication. YH is the principal investigator and is responsible to CMHS. YX, TL, XiuX, YaY, SX, LL, YoY, XiaX and ZW are co-principal investigators who contributed to the design and implementation of the study. ZL, TZ, BW and WG assisted the co-principal investigators in the design, implementation, and data analysis. LW, JY and GX contributed to the study design, implementation of fieldwork, and quality control of collected data. TZ and ZL performed data cleaning, checking and coding. PX analyzed the data for the study, and wrote the initial draft. YH, ZL, BW and WG supervised the analysis and amended the manuscript. All authors have read and approved the final manuscript. The corresponding author attests that all listed authors meet authorship criteria and that no others meeting the criteria have been omitted.

## Data sharing statement

The data of CMHS are not available for sharing.

## Declaration of interests

We declare no competing interests.

## Appendix A Supplementary data

eFigs. 1–3 and eTables 1–5

Translated Abstract
