# Supplementary material for: The prevalence and profiles of adverse childhood experiences and their associations with adult mental health outcomes in China: a cross-sectional study
Source: Lancet Reg Health West Pac. 2024 Dec 5;53:101253. doi: 10.1016/j.lanwpc.2024.101253 (PMC11665606; doi:10.1016/j.lanwpc.2024.101253)
Supplement: eFigs. 1–3 and eTables 1–5 [file mmc1.pdf]

1. Specific Definitions of Adverse Childhood Experiences
2. eTable 1 Fit Indices for Latent Class Models 1–4
3. eFigure 1 Sampling Procedure Flowchart of China Mental Health Survey
4. eFigure 2 The Gender Difference in the Prevalence of Adverse Childhood Experiences
5. eTable 2 The Characteristics of Socio-demographics Variables across Respondents With and Without ACEs Exposure.
6. eTable 3 The Characteristics of Socio-demographics Variables across Four Profiles of ACEs.
7. eTable 4 The Prevalence of Mental Health Outcomes According to Different ACEs.
8. eFigure 3 Gender Difference in Association Between ACEs and Mental Health Outcomes (OR (95%CI))
9. eTable 5 Gender Difference in Association Between Multiple ACEs and Mental Health Outcomes (OR (95%CI)

Specific Definitions of Adverse Childhood Experiences

i. **Parental death:** the death of the respondent's biological father or mother during childhood.

ii. **Parental divorce:** the divorce of the respondent's biological parents during their childhood.

iii. **Other parental loss:** separation from the family for more than six months during their childhood, including living with other relatives, residing in a foster home, attending a boarding school, being hospitalized, or staying in a juvenile detention center.

iv. **Caregiver's mental disorder:** either of caregivers suffering from mood disorder, generalized anxiety disorder, panic disorder during respondent's childhood.

v. **Caregiver's substance abuse:** either of caregivers with a history of alcohol abuse or drug use during respondent's childhood.

vi. **Caregiver's criminality:** either of caregivers had been involved in criminal activities, such as theft or trafficking, or had been arrested and imprisoned during respondent's childhood.

vii. **Caregiver's violence:** either of caregivers frequently involved in physical fight during respondent's childhood.

viii. **Physical abuse:** high frequency of slapped, hit, pushed, grabbed, shoved or threw something at them at home during respondent's childhood.

ix. **Sexual abuse:** any instance where the respondent was subjected to sexual intercourse, or where a finger or object was inserted into their body against their will during childhood.

x. **Neglect:** high frequency of having inadequate food, clothing, or medical care and supervision despite these resources being available, and having to do age-inappropriate chores during childhood.

xi. **Severe physical illness:** experiences of life-threatening illnesses during childhood.

xii. **Family economic adversity:** family received money from a governmental assistance program exceeding six months during the respondent's childhood.

eTable 1 Fit Indices for Latent Class Models 1–4

Number of class	Log-likelihood	G-squared	AIC	BIC	SSABIC	Entropy
2	-13891.32	1094.88	1144.88	1323.53	1244.08	0.66
3	-13642.33	596.90	672.90	944.45	823.69	0.78
**4**	**-13559.74**	**431.71**	**533.71**	**898.16**	**736.09**	**0.82**
5	-13530.41	373.05	501.05	958.40	755.02	0.82

AIC: Akaike Information Criterion; BIC: Bayesian Information Criterion; SSABIC: sample size adjusted BIC

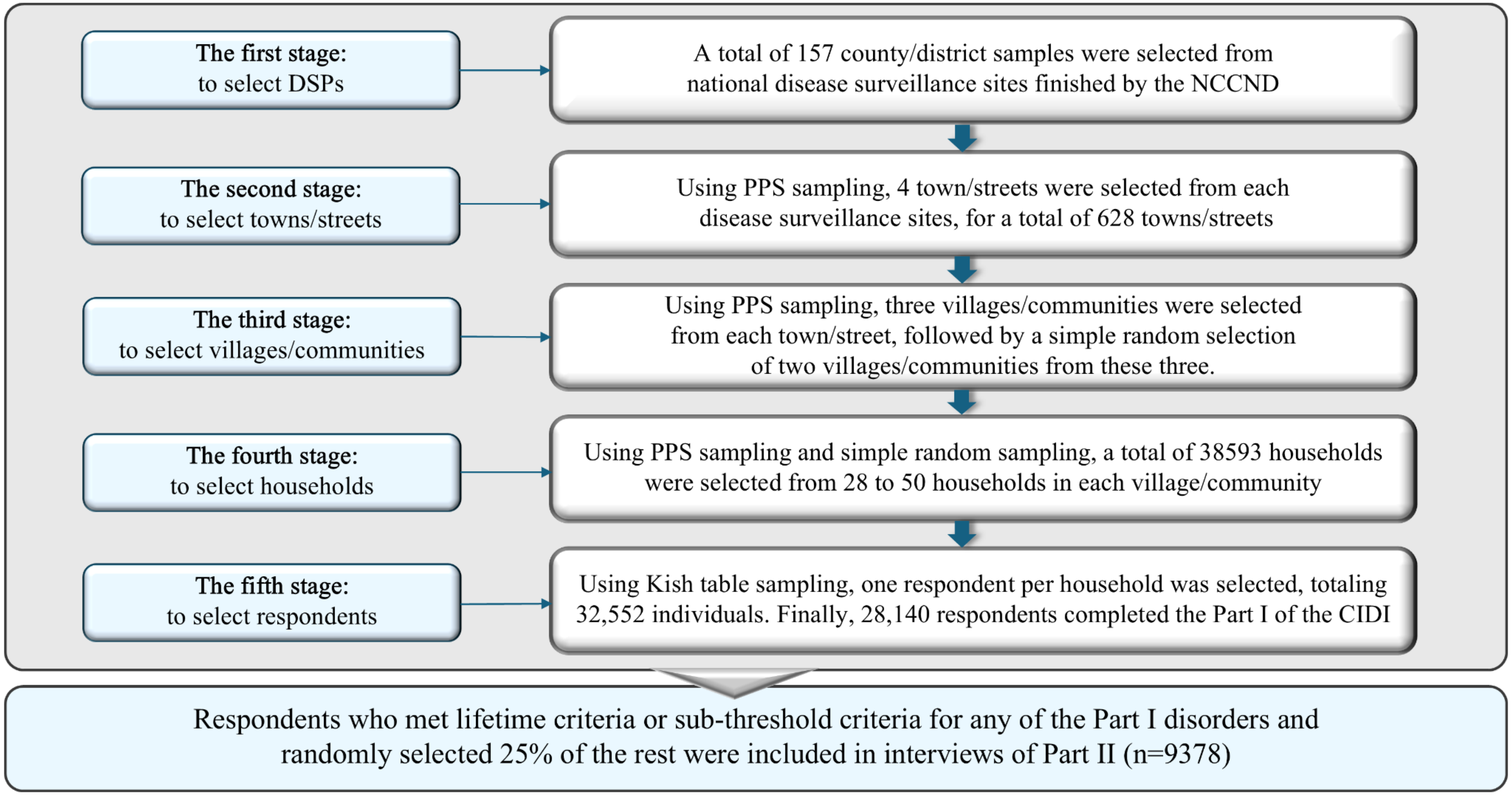

eFigure 1 Sampling Procedure Flowchart of China Mental Health Survey

NCCND: National Centre for Chronic and Non communicable Disease Control and Prevention; DSP: Disease Surveillance Point; CIDI: Composite International Diagnostic Interview; PPS: Probability Proportionate to Size Sampling

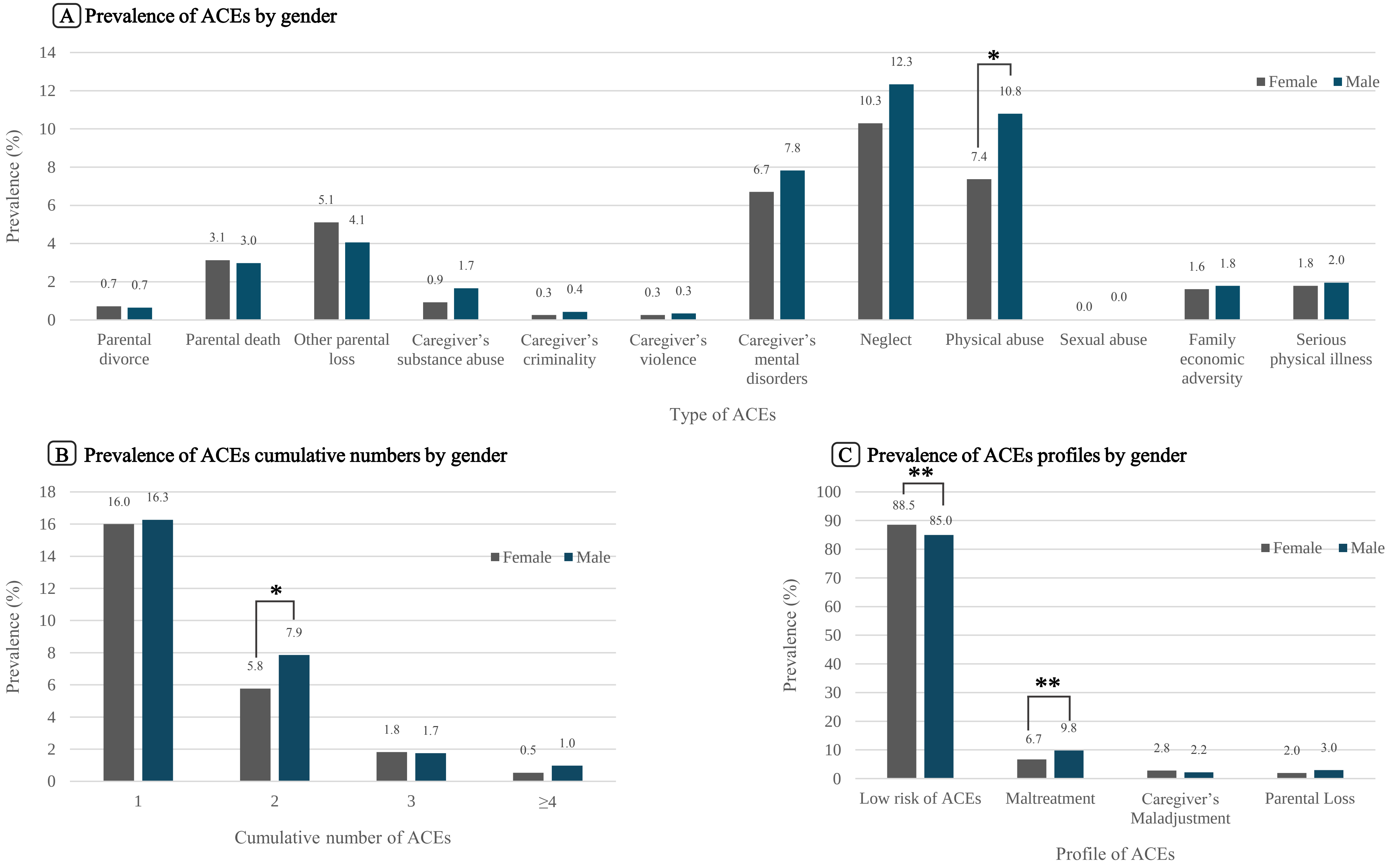

eFigure 2 The Gender Difference in the Prevalence of Adverse Childhood Experiences

****p*<0.05 ***p*<0.01**

eTable 2 The Characteristics of Socio-demographics Variables across Respondents With and Without ACEs Exposure.

Socio-demographics variable	Sample NO.† (%)	ACEs exposure With	ACEs exposure Without	P value*
Gender				0.103
Female	4642 (49.5%)	1192 (46.9%)	3449 (50.5%)	
Male	4736 (50.5%)	1349 (53.1%)	3388 (49.5%)	
Age				
Mean±SD	43.0±15.8	45.1±15.2	42.1±16.0	
Marital Status				0.005
Married/Cohabit	7861 (83.8%)	2163 (85.1%)	5698 (83.3%)	
Never married	503 (5.4%)	166 (6.6%)	337 (4.9%)	
Previously married	1014 (10.8%)	212 (8.3%)	803 (11.7%)	
Source				0.889
Rural	4523 (48.2%)	1219 (48.0%)	3304 (48.3%)	
Urban	4855 (51.8%)	1322 (52.0%)	3533 (51.7%)	
Years in education				
Mean±SD	7.7±5.6	7.1±4.9	7.9±5.9	
Region				0.146
Eastern	3742 (39.9%)	911 (35.8%)	2831 (41.4%)	
Central	2863 (30.5%)	838 (33.0%)	2025 (29.6%)	
Western	2773 (29.6%)	792 (31.2%)	1981 (29.0%)	
Working status				0.013
Working	5012 (53.5%)	1244 (48.9%)	3768 (55.2%)	
Unemployed/homemaker/retired	4358 (46.5%)	1297 (51.1%)	3061 (44.8%)	
Ethnic group				0.976
Han	8216 (88.4%)	2225 (88.3%)	5992 (88.4%)	
Non-Han	1081 (11.6%)	294 (11.7%)	787 (11.6%)	

Abbreviations: ACEs, adverse childhood experiences; SD, standard deviation.
†Counts and percents reported in this table were weighted.
*Differences for continuous variables were analyzed using ANOVA, and for categorical variables using the chi-square test.

eTable 3 The Characteristics of Socio-demographics Variables across Four Profiles of ACEs.

Socio-demographics variable	Profile 1 † Low risk of ACEs N=8137	Profile 2 Maltreatment N=734	Profile 3 Caregiver maladjustment N=233	Profile 4 Parental loss N=234	*P* value*	Pairwise Comparisons
Gender						
Female	4110 (50.5%)	309 (40.0%)	92 (39.3%)	131 (55.7%)		
Male	4026 (49.5%)	464 (60.0%)	142 (60.7%)	104 (44.3%)	0.002	2>4; 3>1,4
Age						
Mean±SD	42.7±15.9	41.6±14.6	45.0±14.2	53.7±14.8	<0.001	4>1,2,3
Marital Status						
Married/Cohabit	6803 (83.6%)	660 (85.4%)	195 (83.7%)	202 (86.0%)		
Never married	423 (5.2%)	41 (5.3%)	13 (5.5%)	26 (10.9%)	0.032	4>1
Previously married	910 (11.2%)	72 (9.3%)	25 (10.8%)	7 (3.0%)		
Source					0.336	
Urban	3963 (48.7%)	357 (46.2%)	91 (38.8%)	112 (47.7%)		
Rural	4174 (51.3%)	417 (53.8%)	143 (61.2%)	123 (52.3%)		
Years In Education						
Mean±SD	7.7±5.8	7.9±4.4	7.2±3.0	5.2±3.9	<0.001	1,2>3>4
Region					0.341	

Eastern	3298 (40.5%)	296 (38.2%)	82 (35.0%)	67 (28.6%)		
Central	2457 (30.2%)	221 (28.5%)	86 (36.9%)	99 (42.4%)		
Western	2382 (29.3%)	257 (33.3%)	65 (28.1%)	68 (29.1%)		
Working status						
Working	4420 (54.4%)	389 (50.2%)	130 (55.6%)	73 (31.3%)	0.002	1>2,3,4
Unemployed/ homemaker/retired	3708 (45.6%)	385 (49.8%)	103 (44.4%)	161 (68.7%)		
Ethnic group					0.888	
Han	7135 (88.4%)	662 (86.7%)	208 (90.1%)	211 (91.1%)		
Non-Han	936 (11.6%)	102 (13.3%)	23 (9.9%)	21 (8.9%)		

Abbreviations: ACEs, adverse childhood experiences; SD, standard deviation.

*Between-cluster differences for continuous variables were analyzed using ANOVA, and for categorical variables using the chi-square test. Pairwise comparisons among multiple groups were adjusted using the Bonferroni correction.

†Counts and percents reported in this table were weighted.

eTable 4 The Prevalence of Mental Health Outcomes According to Different ACEs.

ACEs	Group	Mood disorder	Anxiety disorder	Substance-use disorder	Suicide
Parental divorce	Without	886 (9.5%)	697 (7.5%)	500 (5.4%)	134 (1.4%)
	With	14 (21.4%)	7 (11.5%)	4 (6.8%)	2 (3.8%)
Parental death	Without	843 (9.3%)	663 (7.3%)	492 (5.4%)	118 (1.3%)
	With	55 (19.4%)	36 (12.6%)	13 (4.6%)	17 (6.0%)
Other parental loss	Without	837 (9.4%)	656 (7.3%)	486 (5.4%)	120 (1.3%)
	With	63 (14.7%)	49 (11.3%)	19 (4.3%)	16 (3.8%)
Caregiver's substance abuse	Without	862 (9.4%)	672 (7.3%)	489 (5.3%)	125 (1.4%)
	With	33 (27.0%)	25 (20.8%)	13 (10.9%)	11 (9.3%)
Caregiver's criminality	Without	885 (9.5%)	691 (7.5%)	496 (5.3%)	135 (1.5%)
	With	9 (28.2%)	6 (19.3%)	7 (21.6%)	0 (0.7%)
Caregiver's violence	Without	884 (9.5%)	692 (7.5%)	495 (5.3%)	134 (1.4%)
	With	10 (35.9%)	5 (17.3%)	8 (27.3%)	1 (5.3%)
Caregiver's mental disorders	Without	685 (8.1%)	534 (6.3%)	381 (4.5%)	106 (1.3%)
	With	174 (26.1%)	144 (21.6%)	110 (16.4%)	26 (3.9%)
Neglect	Without	698 (8.5%)	515 (6.3%)	361 (4.4%)	88 (1.1%)
	With	183 (17.4%)	171 (16.3%)	132 (12.5%)	46 (4.3%)
Physical abuse	Without	719 (8.5%)	565 (6.7%)	383 (4.5%)	95 (1.1%)
	With	178 (21.0%)	131 (15.4%)	121 (14.2%)	41 (4.8%)
Sexual abuse	Without	896 (9.6%)	701 (7.5%)	503 (5.4%)	136 (1.5%)
	With	1 (33.1%)	1 (44.8%)	0 (17.1%)	0 (6.2%)
Family economic adversity	Without	864 (9.4%)	660 (7.2%)	498 (5.4%)	133 (1.5%)
	With	33 (20.7%)	32 (20.2%)	6 (3.6%)	3 (1.8%)
Serious physical illness	Without	848 (9.2%)	671 (7.3%)	480 (5.2%)	125 (1.4%)
	With	52 (29.8%)	33 (18.9%)	25 (14.0%)	11 (6.3%)
Any adverse childhood experiences	Without	444 (6.5%)	341 (5.0%)	234 (3.4%)	50 (0.7%)
	With	456 (17.9%)	364 (14.3%)	271 (10.7%)	86 (3.4%)
Number of ACEs	1	239 (15.1%)	191 (12.1%)	150 (9.4%)	38 (2.4%)
	2	124 (18.0%)	97 (14.2%)	75 (11.0%)	25 (3.6%)
	3	68 (35.5%)	58 (30.2%)	29 (15.2%)	14 (7.2%)

	≥4	25 (33.0%)	18 (22.8%)	17 (21.6%)	10 (13.0%)
Profile of ACEs	Profile 1: Low risk of ACEs	638 (7.8%)	509 (6.3%)	361 (4.4%)	83 (1.0%)
	Profile 2: Maltreatment	148 (19.2%)	104 (13.4%)	110 (14.2%)	32 (4.1%)
	Profile 3: Caregiver's maladjustment	71 (30.5%)	63 (27.1%)	28 (11.8%)	9 (4.0%)
	Profile 4: Parental loss	43 (18.2%)	29 (12.2%)	7 (2.8%)	12 (5.0%)

Counts and percents reported in this table were weighted.
Abbreviations: ACEs, adverse childhood experiences; SD, standard deviation.

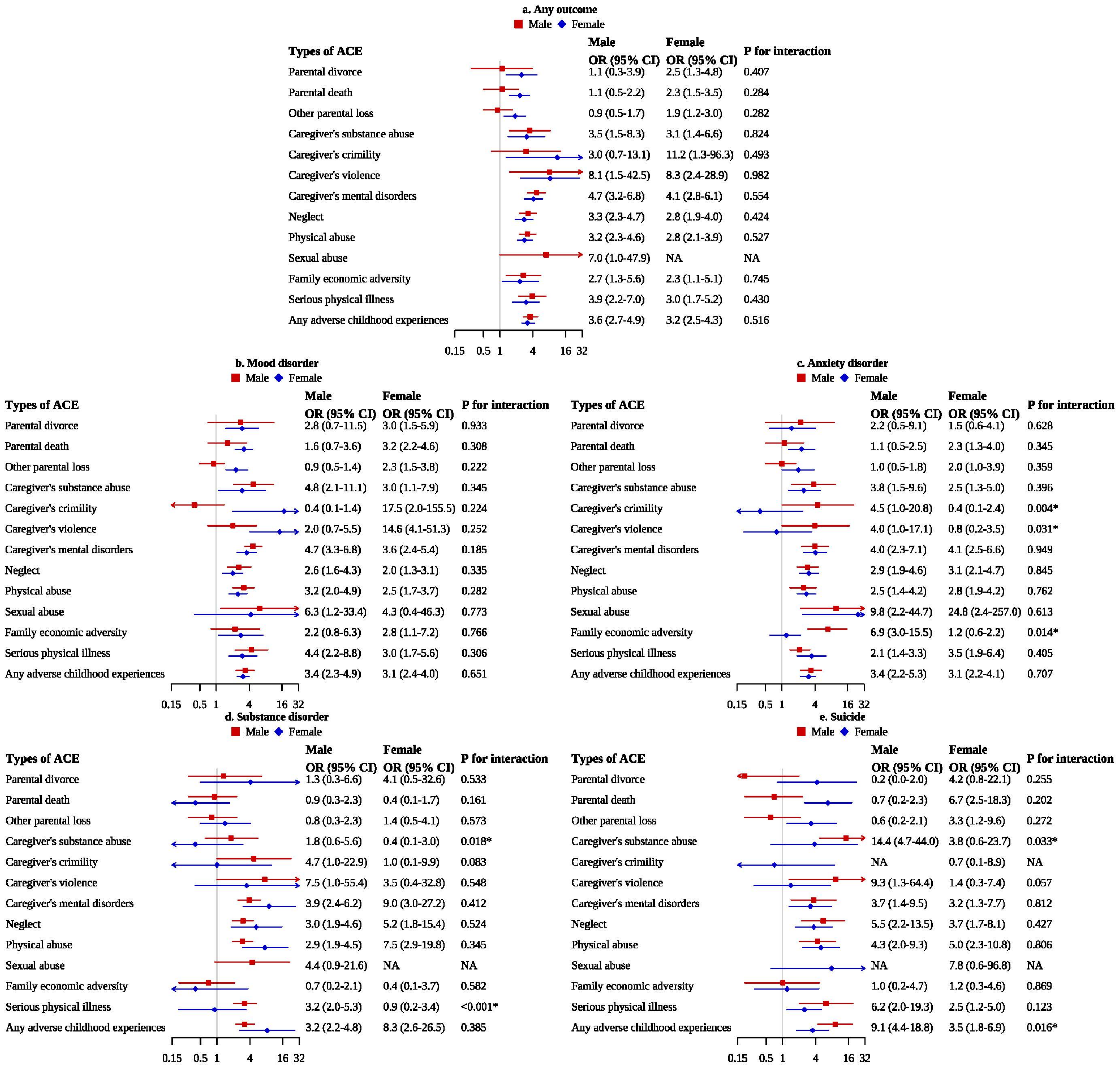

eFigure 3 Gender Difference in Association Between ACEs and Mental Health Outcomes (OR (95%CI)).

Abbreviations: ACEs, adverse childhood experiences; OR, odds ratio; CI, confidence interval.

Models were adjusted for gender, age, rural or urban residence, education level, marital status, region, working status and ethnic group.

Odds ratios (ORs) represent the association between ACEs and mental health outcomes.

Error bars indicate 95%CIs.

eTable 5 Gender Difference in Association Between Multiple ACEs and Mental Health Outcomes (OR (95%CI)).

Type of ACEs	Mood disorder			Anxiety disorder			Substance-use disorder			Suicide			Any outcome		
	Male	Female	*p* for interaction	Male	Female	*p* for interaction	Male	Female	*p* for interaction	Male	Female	*p* for interaction	Male	Female	*p* for interaction
Number of ACEs															
1	2.6 (1.5-4.5)	2.7 (2.1-3.4)	0.943	2.8 (1.7-4.5)	2.5 (1.7-3.5)	0.658	2.9 (1.8-4.7)	6.7 (1.8-25.0)	0.438	6.3 (1.8-21.9)	2.3 (1.1-5.1)	0.132	3.0 (2.1-4.3)	2.7 (2.0-3.7)	0.724
2	3.7 (2.3-5.9)	2.7 (1.7-4.5)	0.133	3.5 (2.0-6.1)	3.1 (1.9-5.3)	0.853	3.1 (1.8-5.5)	9.7 (2.0-46.8)	0.380	12.4 (5.1-30.1)	3.3 (1.5-6.9)	0.019	4.2 (2.7-6.3)	3.2 (2.0-5.0)	0.344
3	8.5 (4.2-17.1)	8.6 (5.3-13.8)	0.840	10.0 (4.3-23.3)	6.8 (4.2-11.0)	0.479	5.3 (3.0-9.2)	15.2 (2.7-87.2)	0.453	17.4 (6.5-46.5)	8.3 (3.0-22.7)	0.281	8.0 (4.3-14.9)	8.9 (5.3-14.8)	0.640
4	5.8 (2.2-15.2)	10.1 (3.8-27.0)	0.561	3.5 (1.4-8.4)	12.2 (4.0-37.6)	0.332	5.9 (2.0-17.6)	15. 7(2.1-114.8)	0.530	17.0 (4.8-59.9)	31.0 (4.9-195.1)	0.596	5.0 (1.7-14.2)	10.8 (2.9-40.1)	0.508
Profiles of ACEs															
Profile 2: maltreatment	3.2 (2.2-4.9)	2.3 (1.5-3.3)	0.045	2.3 (1.4-3.8)	2.4 (1.5-3.9)	0.941	3.0 (1.9-4.8)	7.8 (2.7-22.1)	0.385	7.4 (3.0-18.6)	2.6 (1.3-5.1)	0.038	3.3 (2.3-4.7)	2.7 (1.9-3.8)	0.369
Profile 3: caregiver's maladjustment	6.3 (3.3-12.0)	3.9 (2.2-6.9)	0.158	6.1 (3.1-11.8)	4.4 (2.4-8.3)	0.472	2.4 (1.0-5.6)	3.3 (0.9-11.9)	0.600	2.8 (0.7-10.8)	5.5 (1.5-19.8)	0.601	5.5 (3.1-9.8)	4.1 (2.4-7.0)	0.368
Profile 4: parental loss	1.4 (0.7-3.0)	3.4 (2.0-5.7)	0.263	1.8 (0.8-4.2)	2.2 (1.3-3.5)	0.594	0.5 (0.2-1.1)	2.4 (0.6-9.7)	0.284	2.2 (0.7-7.0)	4.7 (1.9-11.2)	0.456	1.2 (0.6-2.3)	2.5 (1.5-4.2)	0.267

Abbreviations: ACEs, adverse childhood experiences; OR, odds ratio; CI, confidence interval.

Models were adjusted for age, rural or urban residence, education level, marital status, region, working status and ethnic group.

[a] Reference: No ACE exposure.

†Reference: Profile 1 (Low risk of ACEs)
