# Supplementary material for: The prevalence and profiles of adverse childhood experiences and their associations with adult mental health outcomes in China: a cross-sectional study
Source: Lancet Reg Health West Pac. 2024 Dec 5;53:101253. doi: 10.1016/j.lanwpc.2024.101253 (PMC11665606; doi:10.1016/j.lanwpc.2024.101253)
Supplement: Translated Abstract [file mmc2.docx]

This translation in Chinese was submitted by the authors and we reproduce it as supplied. It has not been peer reviewed. Our editorial processes have only been applied to the original abstract in English, which should serve as reference for this manuscript.

目的

童年负性经历普遍发生并且与精神障碍密切关联。然而，中国成人的童年负性经历检出率、共存特征及其与不良精神健康结局的关联尚未得到充分研究。

方法

中国精神卫生调查（China Mental Health Survey，CMHS）作为中国首次具有全国代表性的精神障碍流行病学调查，于2013年7月至2015年3月间开展，共纳入了28,140名成年受访者。研究采用复合国际诊断交谈表（CIDI）3.0，在包含9,378名受访者的代表性子样本中调查了心境障碍、焦虑障碍、物质使用障碍及自杀相关行为四类不良精神健康结局。通过潜类别分析探索中国成人童年负性经历的共存特征；应用logistic回归分析童年负性经历与不良精神健康结局的关联，并通过计算人群归因分值量化童年负性经历对不良精神健康结局的贡献。

结果

9378名受访者的加权后平均年龄为43.0岁，女性加权比例为49.50%。研究发现，27.1%的受访者报告了至少经历过一种童年负性事件；而在有童年负性经历的受访者中，37.6%的人报告同时经历了多种童年负性事件；忽视是最常见的童年负性经历类型（11.3%），其次是躯体虐待（9.1%）。潜类别分析确定了中国成人童年负性经历的四种共存模式：低水平童年负性经历类型、虐待类型、抚养人角色适应不良类型和父母关系缺失类型。抚养人角色适应不良类型与不良精神健康结局的关联最强（OR，4.9；95% CI，3.2-7.6）。所有童年负性经历共同解释了39.4%（95%CI，31.3%-47.4%）的本研究关注的不良精神健康结局。此外，在童年负性经历流行率及其与精神健康结局的关联方面存在显著的性别差异。

结论

中国成人的童年负性经历检出率较高且普遍相互关联，与超过三分之一的心境障碍、焦虑障碍、物质使用障碍及自杀相关行为有关。在资源有限的条件下，探索并干预多种童年负性经历的潜在共因，并通过教育和政策优先预防最常见和影响最大的童年负性经历类型可能是减轻精神疾病负担的有效策略。
